# Study of Concrete Deterioration Damage by Landfill Leachate in Cold Regions

**DOI:** 10.3390/ma18102361

**Published:** 2025-05-19

**Authors:** Yuejia Chen, Mengya Wang, Tiefu Xu, Jinsuo Liu, Zijun Zang, Siru Li, Xuebin Jia, Jialu Ma

**Affiliations:** 1 School of Civil Engineering, Heilongjiang University, Harbin 150080, China; 2016072@hlju.edu.cn (Y.C.); wmy10130915@163.com (M.W.); liujinsuo0909@163.com (J.L.); 17845079010@163.com (Z.Z.); 15903721789@163.com (S.L.); 2009030@hlju.edu.cn (X.J.); 2 Institute of Engineering Mechanics, China Earthquake Administration, Harbin 150080, China; jialuma@163.com

**Keywords:** cold regions, landfill, concrete corrosion, multifactor conditions

## Abstract

The corrosion mechanism of concrete structures in landfills in cold regions is complex, and there are few existing studies that address multifactorial coupled deterioration scenarios. Since loading and freeze-thaw cycles affect concrete deterioration, this study included three test groups—landfill leachate, loaded-landfill leachate, and freeze-thaw cycles-loaded-landfill leachate—and three different corrosion scenarios—gas-liquid, liquid-solid, and gas-liquid-solid. The physico-mechanical changes in concrete in terms of mass, compressive strength, and dynamic elasticity modulus were analyzed, and the deterioration mechanism of concrete was elaborated by its apparent morphology and scanning electron microscope (SEM) images. The study showed that the most serious damage to concrete was caused by freeze-thaw cycles, loading, and landfill leachate coupled in multifactorial situations. The compressive strength and dynamic elastic modulus decreased; the endpoints decreased by 15.75% and 7.42%, respectively, and increased by 12.51% and 6.74% compared with the unapplied load group. The concrete in the gas-liquid-solid test group had the most serious damage among the corrosion scenarios, with a 21.63% decrease in compressive strength. This study determined the most unfavorable corrosion conditions for concrete structures in landfills in cold regions and the corrosion mechanism of concrete exposed to landfill leachate and provides a technical reference for the construction of landfill facilities.

## 1. Introduction

Landfills are the most common method of disposing of municipal solid waste (MSW) in many countries [1], and globally 49.6% of MSW is landfilled every year [2]. In China, for example, there were still 1211 sanitary landfills for MSW in China as of 2023 [3], and most landfills generate large quantities of leachate throughout their life cycle, with each ton of MSW generating approximately 0.2 m^3^ of leachate [4]. High concentrations of ammonia nitrogen and low proportions of heavy metals and other organic compounds in landfill leachate result in leakage and will contaminate soil, surface water, and groundwater ecosystems, and ultimately jeopardize human health if not properly managed [5,6]. The presence of chloride (Cl^−^), sulfate (${SO}_{4}^{2-}$), and other ions [7] in the leachate can cause irreversible corrosion of structures, with reactions characterized by high crystallization pressures, which may cause severe damage to the cement matrix [8], ultimately shortening their service life [9,10]. Especially in cold regions, the factors affecting the deterioration damage of concrete structures in landfills are very complex, and simulations of the actual working conditions to explore the influence mechanism are lacking in current research.

The factors of deterioration damage to concrete structures in landfills are diverse, and related studies have found that landfill leachate, loading, freeze-thaw cycles, and salt solutions all affect concrete deterioration [11,12,13,14]. Wei [15] studied the erosion of mechanical properties of concrete by landfill leachate. Petryna et al. [16] investigated the time-varying reliability assessment of concrete structures’ deterioration under fatigue loading. Chen et al. [17] developed a new predictive model for the chloride diffusion coefficient based on mesoscopic simulation and statistical methods, which contributes to durability design, service life prediction, and refinement of chloride migration models for reinforced concrete structures. Yin et al. [18] developed an integrated macro-micro model to characterize the deterioration mechanisms of concrete under sulfate exposure. In cold regions, freeze-thaw cycles are typically the main factor of concrete deterioration [19]. Wang et al. [20] developed a composite freeze-thaw damage model for modified recycled concrete to predict freeze-thaw damage evolution. Huang et al. [21] investigated the mechanical properties and microstructure of concrete of different cement types under freeze-thaw cycles based on multi-scale simulations. In the actual service environment, the factors of concrete deterioration are more complex, with freezing and thawing affecting the pore water state and, in turn, the ion transport [22]. Wei et al. [23] investigated the mechanical properties and pore structure degradation of recycled concrete subjected to sulfate and freeze-thaw cycles. Huang et al. [24] revealed the mechanism of chloride ion migration behavior in concrete subjected to bending loads through experimental and numerical analysis. Sun et al. [25] developed an ontological freeze-thaw damage model to analyze the damage evolution and plastic development of concrete under the coupled effects of loading and freeze-thaw cycles. Dong et al. [26] developed a prediction model for concrete transport performance under the coupled effects of freeze-thaw cycles, high-frequency fatigue load, and chloride erosion. The above studies show that the deterioration damage of concrete involves multiple factors. This deterioration damage becomes more complex when it is exposed to complex environments such as landfill leachate, loading, and freeze-thaw cycles for long periods, an issue that has not been adequately quantified in past studies.

The complexity of concrete deterioration damage in landfills is a complex process and is related not only to various environmental factors but also directly to the corrosive conditions in which it exists. Wang et al. [27] immersed prestressed pipe piles in landfill leachate to simulate the corrosion scenario of concrete immersed in the liquid phase to investigate its durability in harsh environments. Liu et al. [28] conducted a field study of landfills in different regions of China and found that they were facing leachate accumulation in the waste layer and had leachate overflowing the side slopes, and the clogging of the leachate collection system (LCS) and the deterioration of the reverse osmosis lining system could lead to leachate leakage problems [29,30]; contaminated soil can also synergistically corrode concrete structures. Miguel Angel Baltazar-Zamora et al. [31] buried reinforced concrete in highly plastic (MH) soils contaminated with chlorides and simulated a scenario in which the concrete was in contact with the solid phase to evaluate the degree of corrosion of concrete structures caused by the subsurface soil. Yang et al. [32] studied the degradation behavior of concrete in contact with solid-liquid two-phase and solid-phase cycles by cyclically immersing concrete in saline soil with sodium sulfate solution and saline soil without sodium sulfate solution. Wang et al. [33] investigated the physicochemical properties of mortar buried in saline soil and salt solution to compare and analyze the degradation mechanism of mortar in the liquid-solid phase. Currently, most studies on single-phase environments have been conducted, and few studies have investigated the deterioration damage of concrete in landfills in multi-phase environments. However, the concrete of the regulating tanks or other facilities is in direct contact with the leachate in a gas-liquid two-phase environment, and the leachate leakage will indirectly contact the concrete after seeping into the soil, resulting in a solid-liquid two-phase and even a gas-liquid-solid three-phase environment. Therefore, it is necessary to simulate the actual conditions to examine the deterioration characteristics and mechanisms of concrete structures.

Based on the above deficiencies, in this study, to analyze the corrosion damage of concrete structures in landfills in cold regions, three test groups were set up—landfill leachate, loaded-landfill leachate, and freeze-thaw cycles-loaded-landfill leachate—as well as gas-liquid, liquid-solid, and gas-liquid-solid corrosion scenarios. By quantifying the degree of concrete deterioration using physical and mechanical indexes, such as mass, compressive strength, and dynamic elastic modulus, and combining them with the apparent morphology and scanning electron microscope (SEM) images, the deterioration mechanism of concrete was analyzed. Since loading affects concrete deterioration, this study also compares the extent of concrete damage at different stress levels, and the most severe damage to concrete was expected in the freeze-thaw cycles-loaded-landfill leachate and gas-liquid-solid test groups. This study simulates the actual corrosion situation and tries to analyze the deterioration mechanism of concrete, which provides data support and a theoretical basis for studying the deterioration mechanism of concrete structures in landfills in cold regions.

## 2. Materials and Methods

### 2.1. Concrete Specimen Preparation

**Figure 1:**
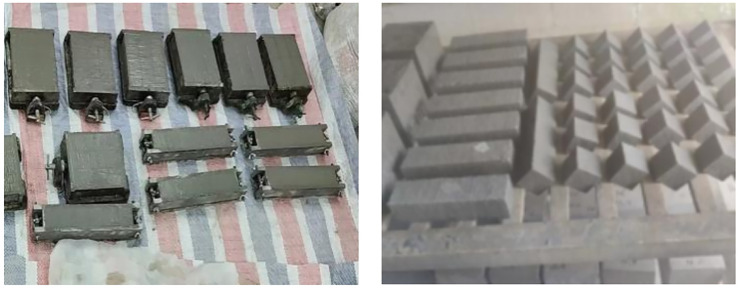
Concrete preparation and curing.

The strength of the concrete used in the tests was C30; a total of 210 cubic specimens (0.04 × 0.04 × 0.04 m^3^) and 105 prismatic specimens (0.04 × 0.04 × 0.16 m^3^) were prepared. The material dosages utilized for the fabrication of concrete specimens are summarized in Table 1. The cement:water:fine aggregate:coarse aggregate:water-reducing agent ratio of the concrete was 1:0.45:1.875:2.875:0.004. Cement (Jidong brand P.O 42.5 ordinary silicate cement) and fine and coarse aggregates were added to the mixer; the coarse aggregate was continuous graded gravel with a particle size of 5–20 mm, and the fine aggregate was natural river sand with a fineness modulus of 2.8. After mixing for 1 min, water and polyhydroxy acid high performance water-reducing agent were added at a consistent rate, and mixing was continued for 5 min. The mixture was transferred into pre-prepared and subjected to vibration compaction on a vibrating table. After the specimens were fabricated, they were demolded after setting for 1 d initially indoors; the demolded specimens were then placed in a standard curing room for 28 d to ensure that they reached the expected strengths and properties [34]. Concrete preparation and curing are shown in Figure 1.

### 2.2. Landfill Leachate

Because Chen et al. [35] demonstrated that young landfill leachate (<5 years) has the most serious corrosive effect on concrete, the landfill leachate used in this test was young landfill leachate from a landfill in Harbin. Samples were taken in batches at different times. The test results for the water quality indicators of the landfill leachate are presented in Table 2.

### 2.3. Test Design

**Figure 2:**
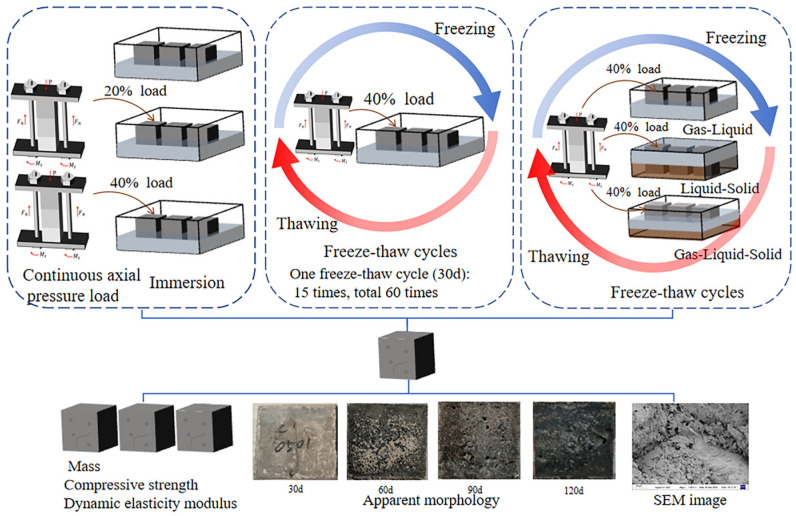
Flow of concrete deterioration damage test under different coupling conditions and corrosion scenarios.

The design of the concrete deterioration damage test under different coupling conditions and corrosion scenarios is presented in Table 3, and the test flow is shown in Figure 2. The test evaluated the application of a continuous axial pressure load to the maintenance of a 28 d compressive strength value as a benchmark. The freeze-thaw cycles, load, and landfill leachate concrete test group are shown after continuous load immersion for 3 d to ensure complete hydration. Freeze-thaw cycle test conditions were based on the local temperature changes in the Harbin set. The mass, compressive strength (YAW-Z300S, Hengruijin Testing Machine Company, Jinan, Shandong, China), and dynamic elastic modulus (DT-20 W, Huiyou Instrument and Equipment Company Limited, Tianjin, China) of the concrete were measured at the end of each test cycle. When the number of freezing and thawing reaches 60 times, the loss of dynamic elastic modulus of 40% or the loss of mass of 5% of any one of the circumstances occurs to stop the test; refer to GB/T50082-2024 [36]. Simulation of different corrosion scenarios was mixed with the landfill leachate and soil in a mixing ratio of 1:4. The planned soil contact height was marked on the concrete and the concrete was placed in the test container. The first addition to the mixed soil was slightly higher than the marking; natural settlement of 1 d was allowed, and the soil above the marking was removed based on the set test group so the landfill leachate at the upper surface of the concrete did not enter. SEM (ZEISS Sigma 300, Oberkochen, Germany) was performed on the specimens at the end of the test.

## 3. Results and Discussion

### 3.1. Concrete Deterioration Damage Under Loaded-Landfill Leachate Coupling

**Figure 3:**
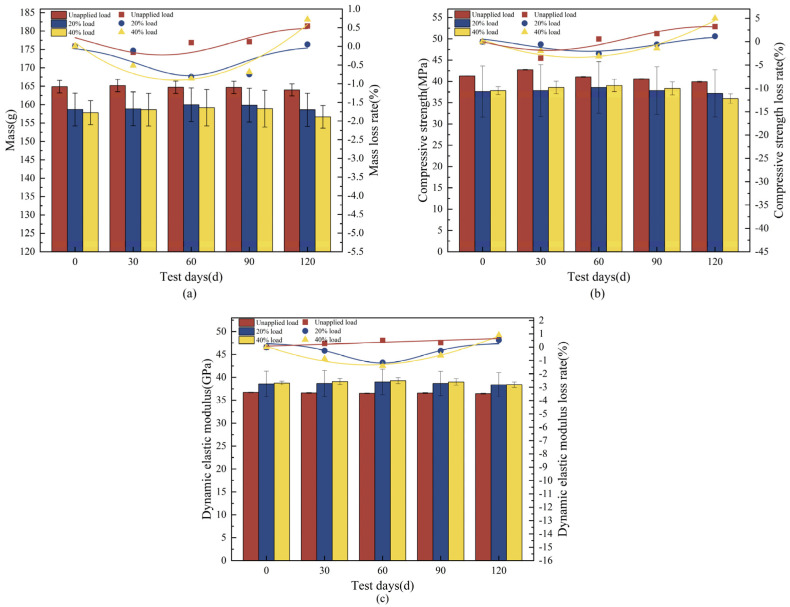
Physical-mechanical index of concrete under load-landfill leachate coupling. (**a**) Mass; (**b**) compressive strength; (**c**) dynamic elastic modulus.

The mass tended to increase and then decrease with the length of the corrosion period (Figure 3a), and the loss of concrete mass for the 20% load group at the endpoint was 0.05%, with mass losses of 0.54% and 0.72% for the unapplied load group and the 40% loaded group, respectively. The mass was slightly less than the initial value for the 20% loaded group, which shows that the proper increase in load has little effect on the mass of concrete. The changes in compressive strength of concrete with 20% and 40% loading were similar, showing an increasing and then decreasing trend (Figure 3b). There was an increase in compressive strength of 2.53% and 3.15%, respectively, during the first 60 d, and a decrease of 1.16% and 4.98%, respectively, at the endpoint. The concrete in the unapplied load group continued to decrease after 30 d of increase, with an endpoint loss of 3.24%, which shows that the loss of compressive strength increased with the application of a higher load percentage. The dynamic elastic modulus varied in a manner similar to the compressive strength (Figure 3c), increasing for the first 60 d and then decreasing until the end of the test, with losses of 0.53% and 0.89%, respectively. At the endpoint, the dynamic elastic modulus in the unapplied load group continued to decrease by 0.68% at the endpoint, and the effect of load application on the dynamic elastic modulus was not significant. The concrete destruction was most severe in the test group with a 40% load coupled with landfill leachate, with a loss of mass, compressive strength, and dynamic elastic modulus of 0.72%, 4.98%, and 0.89%, respectively, which were higher than those in the other two groups.

**Figure 4:**
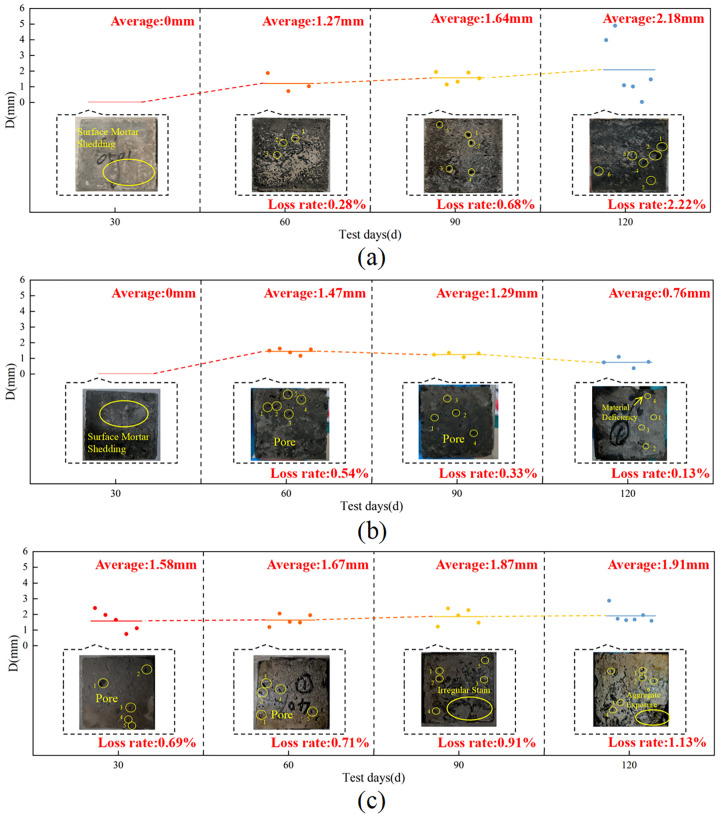
Apparent morphology of the concrete under load-landfill leachate coupling. (**a**) Unapplied load; (**b**) 20% load + landfill leachate; (**c**) 40% load + landfill leachate.

As the test proceeded, the concrete surface showed different degrees of damage, and at 60 d, the average diameter of the holes on the surface of the specimens in the unapplied load group was approximately 1.27 mm, and the surface damage of the concrete specimen was approximately 0.28% (Figure 4a). The average hole diameter on the surface of the specimen in the group with 20% load applied was approximately 1.47 mm, and the damage was approximately 0.54% (Figure 4b). The average hole diameter on the surface of the specimen in the 40% load group was approximately 1.67 mm, and the damage on the surface of the specimen was approximately 0.71% (Figure 4c). As corrosion proceeded, both the holes and the rate of loss on the concrete surface gradually increased in the unloaded group and the 40% loaded group, whereas both gradually decreased in the 20% loaded group. After 120 d, the mortar on the surface of the specimens in the unapplied load group peeled off, and holes of different sizes covered the surface, with an average diameter of approximately 2.18 mm, and there was a loss of approximately 2.22% of the surface. The holes in the surface of the specimens with 20% load applied were relatively small, with an average of approximately 0.76 mm, causing a loss of approximately 0.13% and a defect of 0.65% at the edge. The concrete aggregate was exposed by applying a 40% load, the average surface hole diameter was 1.91 mm, which caused approximately 1.13% surface damage, and the corners are seriously defective, with a defect rate of 3.91%. The degree of mortar shedding on the surface of the specimens with 20% load applied was significantly less than that of the specimens with unapplied load applied and those with 40% load applied, and there was more damage to the specimens with 40% load applied than to those of the other two groups, which is in agreement with the changes in the mass of the concrete and its compressive strength. Referring to the research analysis of Liu et al. [37], the application of lower stress (20% load) increased the permeability of concrete to a certain extent, enhanced the ability of concrete to resist leachate corrosion, and the mass of concrete did not change significantly. However, higher sustained stress (40% load) accelerated the deterioration damage of concrete and was not conducive to leachate erosion resistance, and the loss of the compressive strength of the concrete increased.

**Figure 5:**
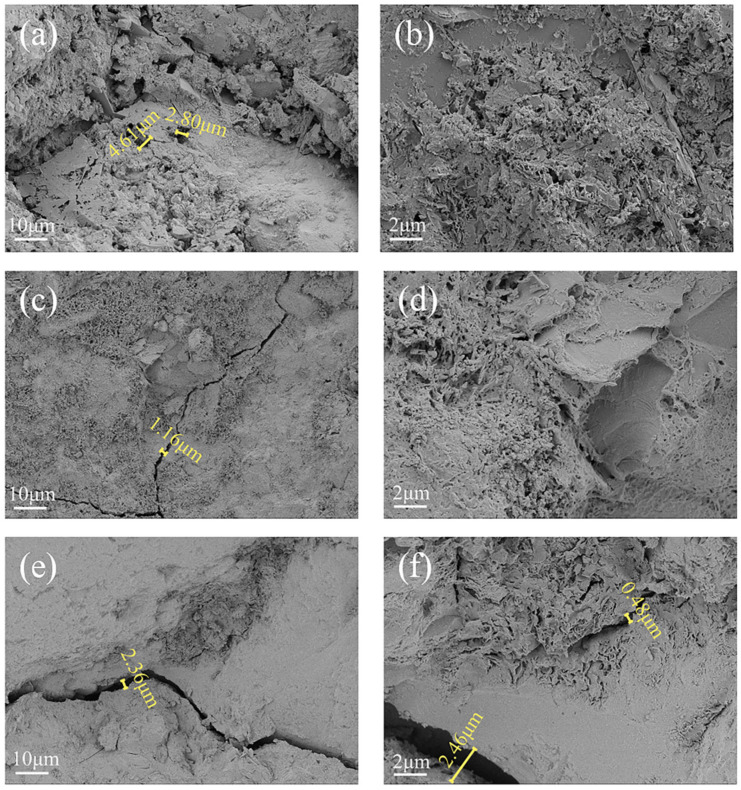
SEM images of concrete under loaded-landfill leachate coupling. (**a**) Unapplied load 1000×; (**b**) unapplied load 5000×; (**c**) 20% load + landfill leachate 1000×; (**d**) 20% load + landfill leachate 5000×; (**e**) 40% load + landfill leachate 1000×; (**f**) 40% load + landfill leachate 5000×.

Referring to the study of Zahoor Hussain et al. [38], the microstructure of concrete after corrosion was carried out using SEM, and according to the results of Figure 5, the interior of the concrete in the unloaded group was internally porous, with holes ranging in size from 2.80 to 4.61 μm (Figure 5a), and the loose structure could be observed at a 5000× magnification (Figure 5b). A 1.16 μm crack appeared in the group with 20% load applied (Figure 5c), which shows that the lower level of load had slightly less effect on the internal cracks of the concrete, and a honeycomb structure was observed in the interior of the concrete at 5000× magnification (Figure 5d), which may be the C-S-H flocculating gel [39]. As the load level increased, the cracks in the applied 40% load group increased to 2.36 μm (Figure 5e), and the surrounding cement matrix was dissolved, indicating that the larger crack width made the area around it more vulnerable to erosion, which is similar to the study by Ying et al. [40]. A crack width of 2.46 μm was observed at 5000× magnification, with overlapping layers of lamellar crystals in which a 0.48 μm crack appeared (Figure 5f). The specimens in the group coupled with 40% loading and landfill leachate were the most significant deterioration, which corresponds with the observed changes in mass, compressive strength, dynamic elastic modulus, and apparent morphology. According to the SEM image analysis, higher sustained loads (40% load) led to crack expansion in the concrete, prompting corrosive ions to react [41]. As erosion proceeded, the hydration product C-S-H calcium leaching increased the porosity and accelerated the migration of ions [42]. In the early stage, the concrete pores are filled with expansion crystals generated by corrosion and become dense. When the original pores were filled, the expansion crystals led to internal expansion stress around the pores of the concrete, so the internal structure was loose, the compressive strength decreased [43], and the coupling of the load and landfill leachate resulted in less increase in compressive strength.

### 3.2. Concrete Deterioration Damage Under Freeze-Thaw Cycles-Loaded-Landfill Leachate Coupling

**Figure 6:**
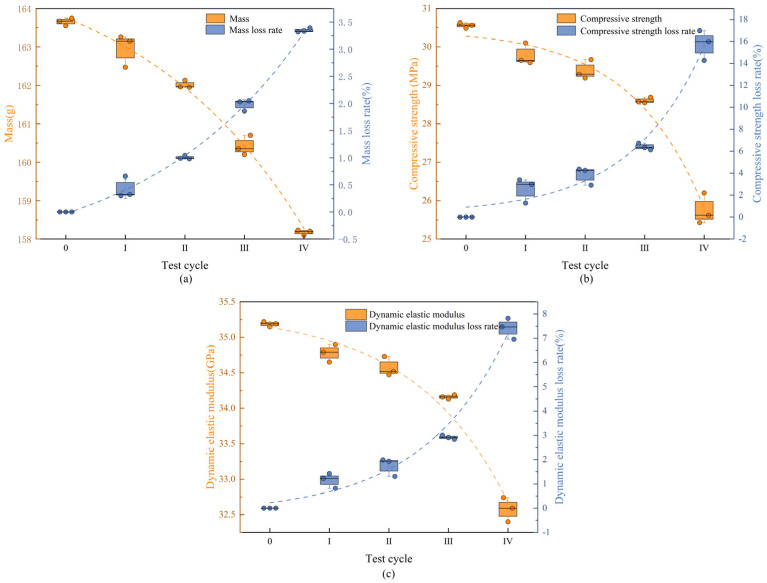
Physical-mechanical indexes under freeze-thaw cycles-loaded-landfill leachate coupling. (**a**) Mass; (**b**) compressive strength; (**c**) dynamic elastic modulus.

Under freeze-thaw cycles, 40% loading, and landfill leachate coupling, the concrete mass showed a decreasing trend with an endpoint mass loss of 3.35% (Figure 6a), which was substantially higher than that of the landfill leachate group (0.54%) and the 40% loading and landfill leachate coupling group (0.72%). The dynamic elastic modulus varied in a manner similar to the compressive strength (Figure 6b,c), with a continuous decrease during the test cycle; the rate of decrease increased with an increase in the number of freezing and thawing cycles, decreasing by 15.75% and 7.42%, respectively, at the endpoint, at which time the concrete was no longer internally dense, and the variation in the modulus of dynamic elasticity was similar to the pattern found by Yu et al. [44]. The deterioration damage of concrete by the coupling of freeze-thaw cycles, 40% load, and landfill leachate is much more significant than that of the landfill leachate group (unapplied load group) and the 40% load and landfill leachate coupling test group; multifactor coupling accelerates the deterioration damage of concrete [45].

**Figure 7:**
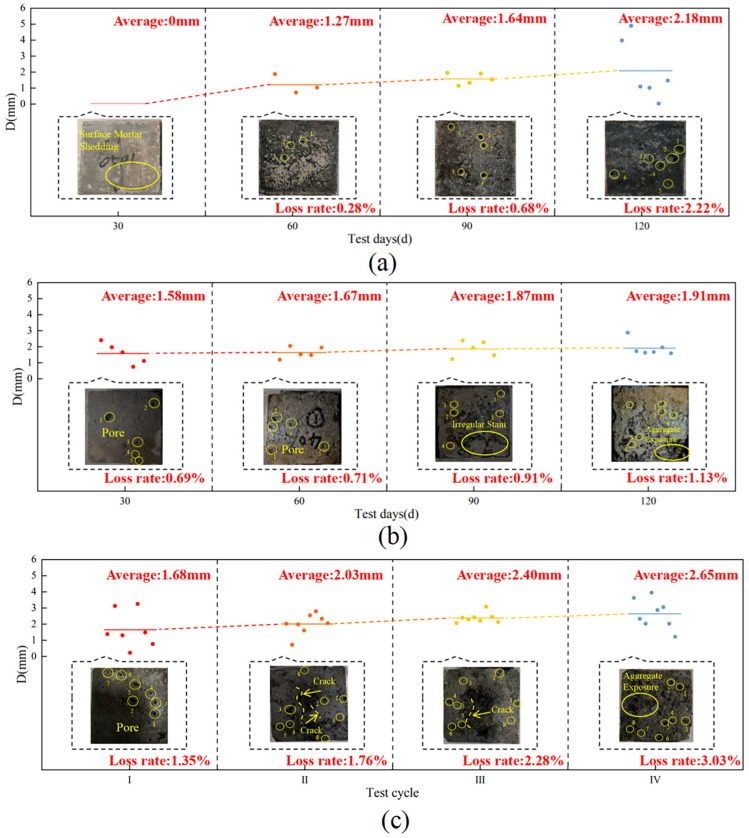
Concrete apparent morphology under different coupling conditions. (**a**) Landfill leachate; (**b**) 40% load + landfill leachate; (**c**) freeze-thaw cycles + 40% loading + landfill leachate.

The apparent morphology of concrete under freeze-thaw cycles, 40% loading, and landfill leachate coupling is shown in Figure 7c. At the end of the I cycle, holes with an average diameter of approximately 1.68 mm appeared on the surface of the concrete with a surface loss of approximately 1.35%. At this time, the number and size of the holes were higher than those of the group with only immersed landfill leachate and the group with applied 40% loading and leachate coupling, and the freeze-thaw cycles exacerbated the formation of the holes. At the end of the II cycle, the holes on the surface of the specimen averaged 2.03 mm, with a surface loss of approximately 1.76%, and cracks appeared. Combined with the analysis of Qiu et al. [46], the accumulation of freeze-thaw cycles led to the enlargement of internal pores in the concrete and the development of cracks between the mortar and the aggregate. The 40% load accelerated the mortar deterioration, which reduced the adhesion between the cementitious material and the aggregate and exacerbated the formation of cracks. The damage was more serious in Cycle III, with holes reaching 2.40 mm and surface loss of approximately 2.28%. At the end of the cycle, the holes were interconnected, resulting in significant surface mortar detachment and exposed aggregates, with an average hole diameter of 2.65 mm and a resultant surface loss of approximately 3.03%.

**Figure 8:**
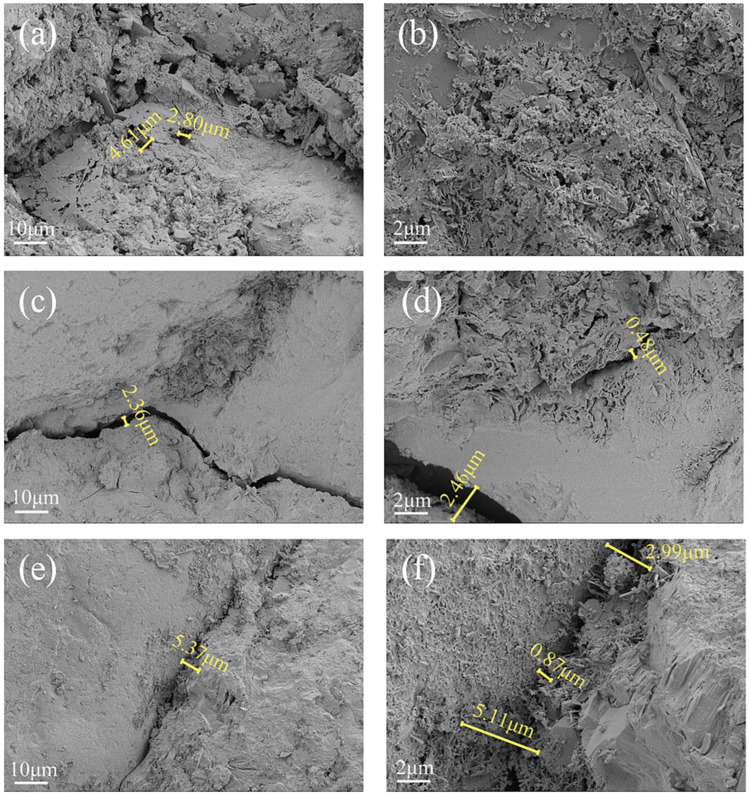
SEM images of concrete under different coupling conditions. (**a**) Landfill leachate 1000×; (**b**) landfill leachate 5000×; (**c**) 40% load + landfill leachate 1000×; (**d**) 40% load + landfill leachate 5000×; (**e**) freeze-thaw cycles + 40% loading + landfill leachate 1000×; (**f**) freeze-thaw cycles + 40% loading + landfill leachate 5000×.

The SEM images of the concrete under different coupling conditions are shown in Figure 8. Cracks of 1.11 μm appeared in the immersed landfill leachate, the applied 40% loading group produced larger 2.46 μm cracks, and in the freeze-thaw cycles, 40% loading, and landfill leachate coupling group, cracks increased and their sizes varied, with widths ranging from 0.87 μm to 5.37 μm (Figure 8e,f). The concrete around the cracks showed a loose and porous structure, and C-S-H gels and rod-like calcite (AFt) crystals [39] were observed growing and distributing at the edge of the cracks at 5000× magnification. The mechanical properties of the concrete were significantly reduced compared to those of the other two groups. Under freeze-thaw cycles, the conversion of pore water into ice caused pore volume expansion, and the resulting tensile stress acted on the pore walls, initiating microcrack formation and propagation [47], coupled with the fact that the 40% higher stress loading produced and expanded the microcracks in the concrete. Cracking caused an increase in permeability [48] and accelerated the infiltration and erosion of corrosive ions in the leachate, while the acidic environment provided by the young leachate causes acidic corrosion and generation of swelling crystals. The coupling of the three factors accelerated the deterioration damage, resulting in a decrease in concrete strength, which was worse than the cases of the landfill leachate group and the applied 40% load coupled with the landfill leachate group.

### 3.3. Concrete Deterioration Damage in Gas-Liquid Two-Phase Corrosion Scenarios

**Figure 9:**
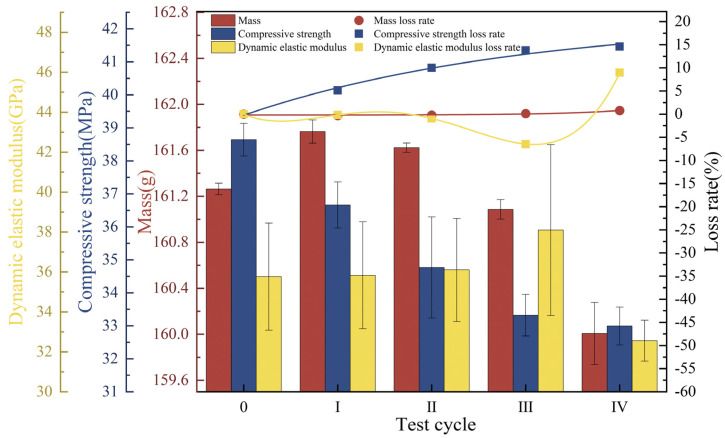
Concrete physico-mechanical indexes under the gas-liquid two-phase corrosion scenario.

The concrete mass increased by 0.31% at the end of Cycle I, and the leachate infiltrated the concrete and hydrated, followed by a decrease in mass and a progressively faster rate of loss, with a loss of 0.78% of mass at the end of the cycle. The compressive strength consistently declined, remaining lower than the initial value throughout, and eventually decreased by 14.61%. The dynamic elastic modulus increased by 6.51% in the first three cycles and decreased rapidly in the last cycle, with an endpoint loss rate of 8.97% (Figure 9).

**Figure 10:**
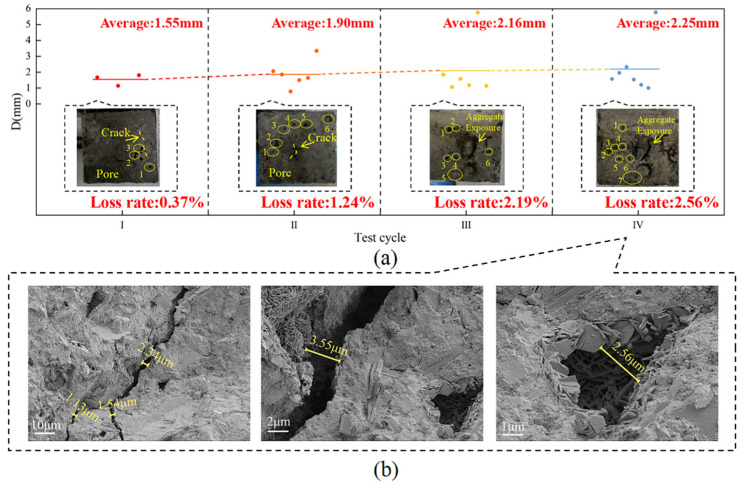
Concrete morphology under the gas-liquid two-phase corrosion scenario. (**a**) Apparent morphology; (**b**) SEM image.

At the end of Cycle I, holes of varying sizes and microcracks appeared on the surface of the concrete, with an average hole diameter of approximately 1.55 mm and a surface loss of approximately 0.37%. In Cycle II, the damage worsened as both the number and size of the pores increased, with an average pore diameter of approximately 1.90 mm and a surface loss of approximately 1.24%. During the freezing process, the difference in ionic concentration in the pore network generates osmotic pressure that leads to cracking of the concrete and the evolution of the pore diameter [49]. At the end of Cycle III, the average surface hole diameter of the concrete specimen was approximately 2.16 mm, causing a surface loss of approximately 2.19% and aggregate spalling at the gas-liquid demarcation. The diameter of the holes increased to 2.25 mm at the end of the test, resulting in a loss of approximately 2.56%, and the color deposition and aggregate spalling at the junction became more severe (Figure 10a), indicating accelerated concrete corrosion at the interface. The SEM image of the concrete is shown in Figure 10b, and it can be observed that there is a crack with a width of 1.13–2.34 μm inside the concrete at 1000× magnification; at 5000× magnification, it can be observed that the width of the crack in the concrete is 3.55 μm and the plate-like crystals on one side of the crack are stacked on top of each other, which may be Friedel’s salt [50]. At 10,000× magnification, it can be observed that the width of the hole is approximately 2.56 μm, surrounded by plate-like crystal (Friedel’s salt), and there are rod-shaped expansion crystals (AFt) of varying lengths irregularly distributed inside. This is due to the instability of the hydration products and chemical reactions with corrosive ions; these reactions reduce the strength of the matrix and thus the mechanical properties [51]. Concrete experiences the most significant damage at the gas-liquid two-phase junction, with internal cracks and holes up to 3.55 and 2.56 μm in width, respectively.

### 3.4. Concrete Deterioration Damage in Liquid-Solid Two-Phase Corrosion Scenarios

**Figure 11:**
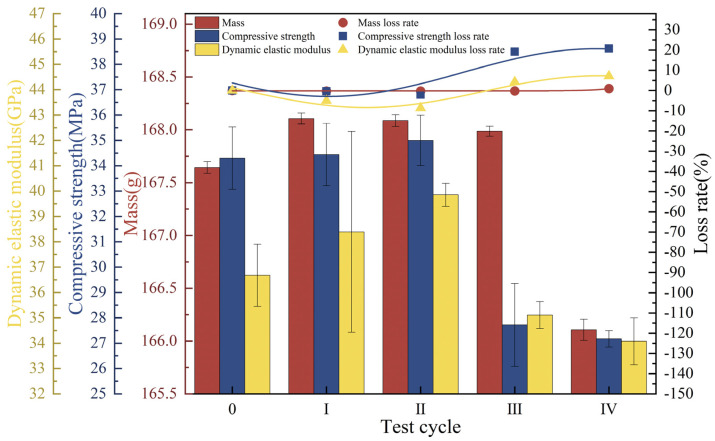
Physical-mechanical indexes of concrete under the liquid-solid two-phase corrosion scenario.

The mass of the concrete specimens first increased and then decreased, with a loss of 0.92% of the endpoint mass. The compressive strength increased by a small amount (2.04%) in the first two cycles and then decreased rapidly, with an endpoint loss of 20.79%. The dynamic elastic modulus increased consistently by 8.67% during the first two test cycles, after which it gradually decreased to 92.91% of its initial value (Figure 11).

**Figure 12:**
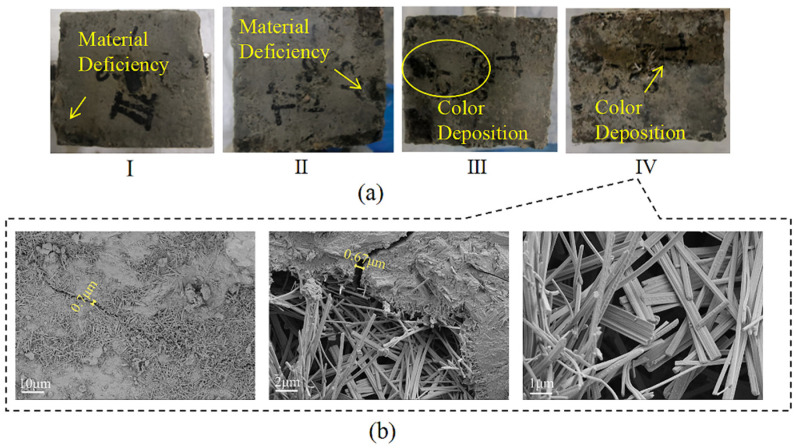
Concrete morphology in the liquid-solid two-phase corrosion scenario. (**a**) Apparent morphology; (**b**) SEM image.

At the end of Cycle I, the concrete surface was no longer smooth, and 3.58% defects appeared at the edges. At the end of Cycle II, the defect rate increased to 4.25%; after that, color buildup on the concrete surface appeared. At the endpoint, color buildup on the portion of the concrete in contact with the landfill leachate was deeper, and the roughness on the surface was more severe than that of the portion of the concrete in contact with the soil (Figure 12a). Referring to the research analysis of Chen [52], the cementitious material is composed of a porous matrix, which is filled with a uniform pore solution, and the ionic concentration gradient in contact with the liquid phase is higher than that of the solid phase. Simultaneously, the corrosive ions in the landfill leachate have a greater rate of diffusion and reaction in the liquid phase than in the solid phase and can more rapidly undergo an erosion reaction in the concrete, which can lead to more serious damage. The SEM image is shown in Figure 12b, and 1000× magnification shows that a crack of 0.7 μm is formed inside the concrete, and rod-shaped crystals (AFt) are distributed around the crack. At 5000× and 10,000× magnification, cracks and holes are forming with a width of approximately 0.67 μm, with a loose concrete matrix around the holes and intertwined internal columnar gypsum [50]. Damage in the liquid-solid phase is more severe when concrete is in contact with the liquid phase than with the solid phase.

### 3.5. Concrete Deterioration Damage in Gas-Liquid-Solid Three-Phase Corrosion Scenarios

**Figure 13:**
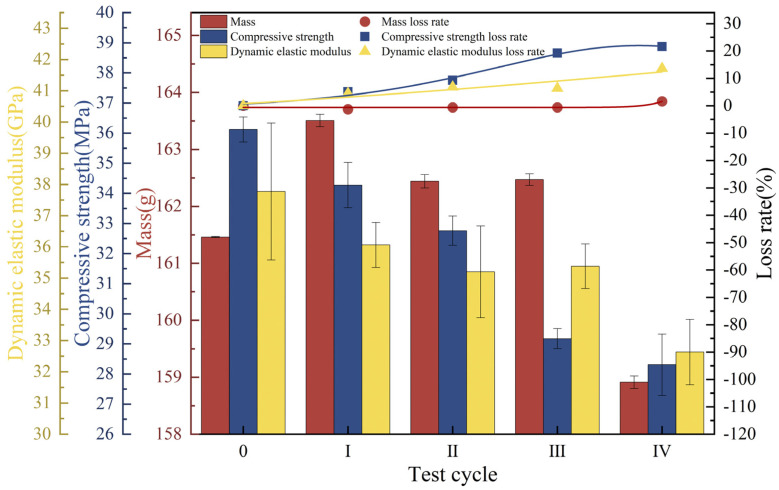
Physical-mechanical indexes of concrete under the gas-liquid-solid three-phase corrosion scenario.

The mass of the concrete specimens increased by 1.27% during Cycle I and continued to decrease after Cycle II, with an endpoint mass loss of 1.58%. The compressive strength was always lower than the initial value and decreased sharply in Cycle III, with the rate of decrease increasing from 0.10 MPa/d to 0.24 MPa/d and slowing down to 0.06 MPa/d in the last cycle, with an endpoint loss rate of 21.63%. The dynamic elastic modulus continued to decrease over the test cycles and eventually decreased by 13.61% (Figure 13).

**Figure 14:**
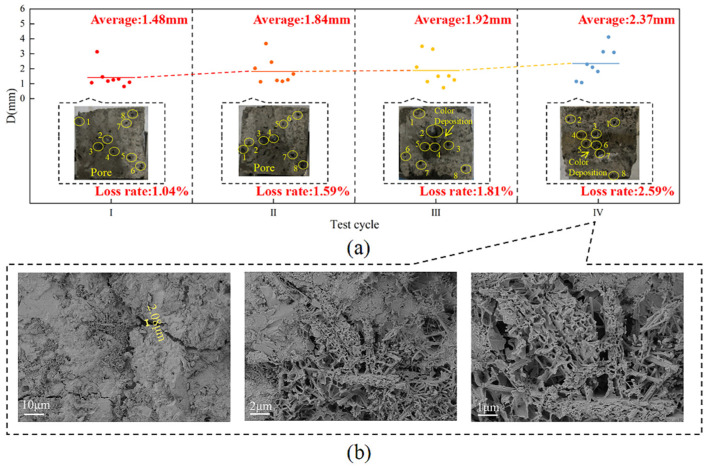
Concrete morphology under the gas-liquid-solid three-phase corrosion scenario. (**a**) Apparent morphology; (**b**) SEM image.

At the end of Cycle I, the average diameter of the holes in the concrete surface was approximately 1.48 mm, causing a surface loss of approximately 1.04%, with 1.91% defects at the upper edge. At the end of Cycle II, the surface roughness of the specimen deepened, and holes with an average diameter of approximately 1.84 mm appeared, with a surface loss of approximately 1.59%. At the end of Cycle III, the average surface pore diameter was approximately 1.92 mm, the surface loss was approximately 1.81%, the color was darker in the area in contact with the landfill leachate, and there was 2.05% aggregate spalling at the lower edge. At the end of the test, the average pore diameter was approximately 2.37 mm, with a surface loss of approximately 2.59%, and there was obvious color stratification on the surface, with a greater change in color at the location in contact with the landfill leachate, as well as white crystals precipitating at the junction of the leachate and the soil (Figure 14a). Combined with the analysis of Yang et al. [53], the formation of corrosion products reduces the pH of the concrete, a concentration difference between the surface and the interior of the concrete occurs, which leaches ions to the pore solution and causes the precipitation of white crystals. Combined with the SEM images of the concrete (Figure 14b), it can be seen that multiple cracks appeared inside the concrete, and a crack of 2.08 μm was observed inside the concrete at 1000× magnification. The concrete matrix around the crack was dissolved, and the densification was no longer good. At 5000× and 10,000× magnification, a large number of flocculent C-S-H gel clumps, interwoven with a small amount of columnar gypsum, could be observed inside the cracks. When concrete is in a gas-liquid-solid three-phase environment, the part in contact with the landfill leachate is the most severely damaged, with crystals precipitating at the junction of the leachate and the soil. The damage to this concrete was the most severe of the three scenarios.

## 4. Conclusions

This study established test groups of landfill leachate, loaded-landfill leachate, and freeze-thaw cycles-loaded-landfill leachate to determine the factors that may cause concrete deterioration and damage in landfill sites in cold regions. In addition, three different corrosion scenarios—gas-liquid, liquid-solid, and gas-liquid-solid—were set according to the different service environments of the concrete in actual working conditions. The appearance, micromorphology, and mechanical properties of the concrete were analyzed, and the following conclusions were drawn:

(1) When load and landfill leachate were coupled, the changes in concrete mass, compressive strength, and dynamic elastic modulus first increased and then decreased. The specimens of the test group that coupled 40% load and landfill leachate had the most serious losses, which were 0.72%, 4.98%, and 0.89%, respectively. The average diameter of the surface holes in the concrete was approximately 1.91 mm, and the spalling was most severe at the edges, with a defect rate of 3.91%. An analysis of the SEM images found that multiple cracks, up to 2.46 μm, appeared inside the concrete.

(2) The trends of three indexes for concrete under the coupled effects of freeze-thaw cycles, 40% loading, and landfill leachate showed a continuous decline, with severe losses of compressive strength and dynamic elastic modulus, which decreased by 15.75% and 7.42%, respectively. The concrete surface exhibited extensive aggregate spalling, and the average hole diameter was approximately 2.65 mm. The freeze-thaw cycles damaged the internal pores of the concrete, and more cracks appeared, with widths ranging from 0.87 to 5.37 μm, and the coupling of the three effects exacerbated the deterioration damage of the concrete.

(3) For the actual environment of concrete in a landfill, three corrosion scenarios were set up from the analysis of the loss of concrete mass, compressive strength, and dynamic elastic modulus. The damage of concrete in the gas-liquid-solid three-phase corrosion scenarios was the most serious, at 1.58%, 21.63%, and 13.61%, respectively, which were higher than those in the gas-liquid and liquid-solid two-phase corrosion scenarios. The average value of the holes on the concrete surface was 2.37 mm, and the SEM image showed that cracks with a width of 2.08 μm appeared inside the specimen, with a large number of corrosion products at the cracks.

This study found that the most serious corrosion of concrete structures is caused by coupled landfill leachate loading and freeze-thaw cycles in cold regions, and the structures in the landfill in the gas-liquid-solid three-phase state should also be a topic of focus. In this study, the mechanism of concrete deterioration under actual working conditions was analyzed and quantified, but there are still limitations, there are differences between the set conditions and the actual changes, and the reactions involving microorganisms in leachate were not considered, and it is also necessary to continue to expand the study of corrosive situations in the future, such as the corrosive effect of contaminated soil freezing and thawing cycles on concrete. This study provides a theoretical basis for the corrosion mechanism of concrete structures in landfills in cold regions and a technical reference for the construction and operation of future landfill facilities.

## Figures and Tables

**Table 1:** Concrete specimen material dosage.

Material Dosage (kg/m^3^)				
Cement	Water	Fine Aggregate	Coarse Aggregate	Water-Reducing Agent
409.32	184.19	767.48	1176.80	1.64

**Table 2:** Test results for water quality indicators of landfill leachate.

pH	c(COD) (mg/L)	c(NH_3_-N) (mg/L)	c(${SO}_{4}^{2-}$) (mg/L)	c(Cl^−^) (mg/L)	c(SS) (mg/L)
5.92–6.24	24,912.36–26,633.60	604.10–664.50	761.75–828.00	541.56–595.56	411.82–452.00

**Table 3:** Concrete deterioration damage tests under different coupling conditions and corrosion scenarios.

Test Group	Tests		Continuous Axial Pressure Load + Landfill Leachate Immersion	Freeze-Thaw Cycles (120 d)			
				I	II	III	IV
1	Loaded-landfill leachate		0 + immersion 120 d	0	0	0	0
			20% load + immersion 120 d	0	0	0	0
			40% load + immersion 120 d	0	0	0	0
2	Freeze-thaw cycles-loaded-landfill leachate		0 + immersion 120 d	0	0	0	0
			40% load + immersion 120 d	0	0	0	0
			40% load + immersion 120 d	30	30	30	30
3	Corrosion scenario	Gas -Liquid	40% load + immersion 120 d	30	30	30	30
		Liquid -Solid	40% load + immersion 120 d	30	30	30	30
		Gas-Liquid-Solid	40% load + immersion 120 d	30	30	30	30

## Data Availability

Data is contained within the article or Supplementary Materials.

## Supplementary Materials

The following supporting information can be downloaded at: https://www.mdpi.com/article/10.3390/ma18102361/s1, Figure S1: Schematic diagram of axial load application device; Figure S2: Three corrosion scenarios settings.
