# Supplementary material for: Study of Concrete Deterioration Damage by Landfill Leachate in Cold Regions
Source: Materials (Basel). 2025 May 19;18(10):2361. doi: 10.3390/ma18102361 (PMC12113290; doi:10.3390/ma18102361)
Supplement: Supplementary file 1 [file materials-18-02361-s001.zip › Supporting information.pdf]

1. Axial load application device

A continuous axial compressive load was applied to the specimen and the device is shown in Figure S.

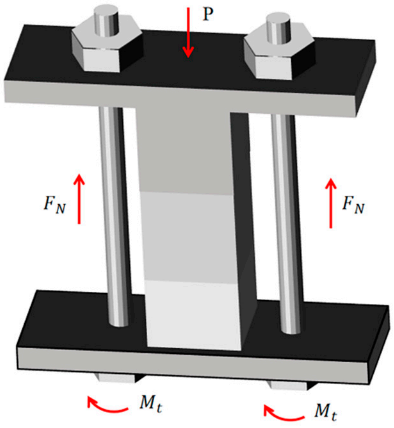

Figure S1. Schematic diagram of axial load application device.

Analyzing the forces on the structure of the device can be obtained:

$$M_t = 0.2F_N \cdot \mathrm{D} \tag{S1}$$

M_t—Torsion(M);

F_N—Screw preload;

D—Bolt Diameter(mm).

The force analysis can be obtained,2F_N=P.According to Equation S1, it can be obtained: bolt prestressing force P=M_t,and the corresponding proportional load is applied using a torque wrench.

2. Corrosive scenario setting

The gas-liquid phase of the setup is commonly found in landfill leachate regulation tanks, the solid-liquid phase is commonly found in collection ponds that are buried and leachate leaks into the soil, and the gas-liquid-solid phase is commonly found in landfill leachate leak into storm drains and nearby soil. The three corrosion scenarios were set up as shown in Figure S.

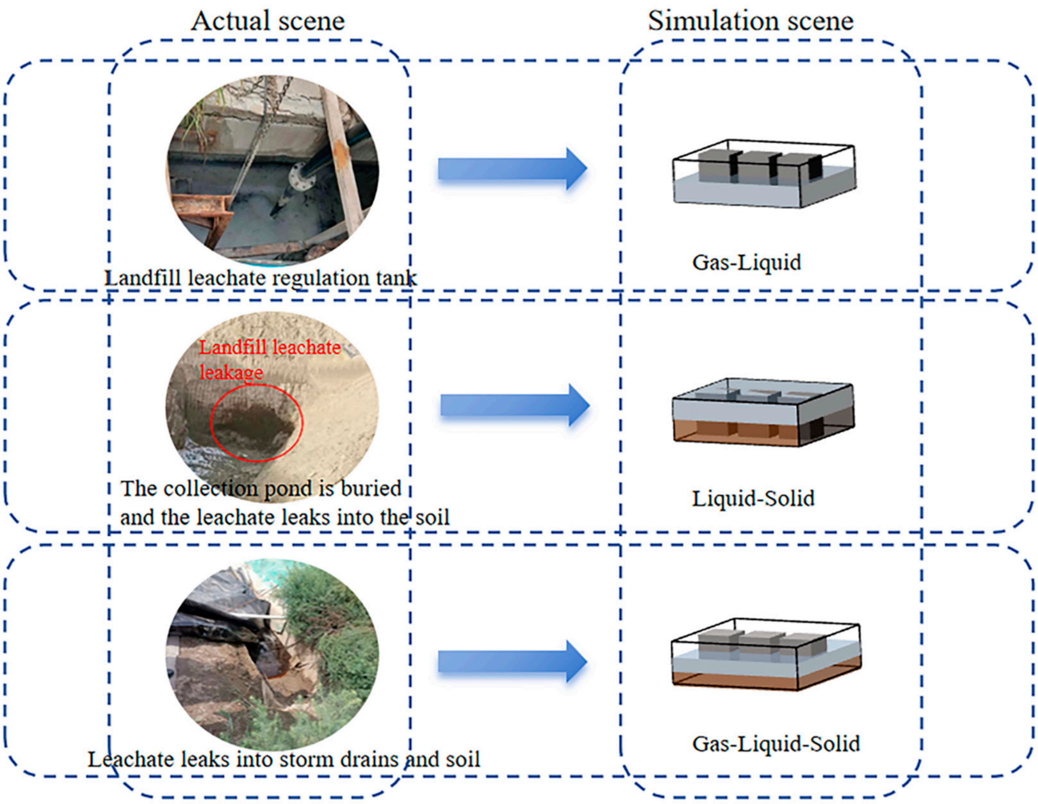

Figure S2. Three corrosion scenarios settings.

3. SEM

Concrete specimens from each test cycle were sampled and the micro-morphology of the concrete at the end of the test cycle was characterized using SEM (ZEISS Sigma 300), made into small specimens of approximately 0.5 mm^3 in size, dried in an oven at 50°C for 12 hours and immersed in anhydrous ethanol to stop hydration, and then observed after the surface was sprayed with gold in vacuum conditions.

4. Test measurement indicators

(1) Mass loss rate

Concrete specimens prior to testing were weighed using a laboratory electronic scale with an accuracy of 0.01 g. At the end of each test cycle, the specimens were removed from surface adhesion and weighed. The arithmetic mean of three concrete specimens was selected as the measured value for each test group and the mass loss rate was used to express the change in mass of the concrete specimens. This can be calculated using Equation S2.

$$\Delta W_r = \frac{(W_0 - W_n)}{W_0} \times 100\% \tag{S2}$$

ΔW_r—Rate of mass loss (%) of concrete specimens after n test cycles, accurate to 0.01;

W_0 —Initial mass of concrete specimens(g);

W_n —Mass of the concrete specimen after n test cycles(g).

(2)Compressive strength loss rate

Take the arithmetic mean of the three specimens as the strength value of the specimen group, accurate to 0.1 MPa. If the difference between the maximum or minimum of the three measured values and the median is greater than 15% of the median, the median is the measured value, and if the difference between the maximum and minimum of the three measured values and the median is greater than 15% of the median, the test result of the group is invalid. Using the rate of loss of compressive strength to determine the change in compressive strength of concrete.This can be calculated using Equation S3.

$$\Delta f_c = \frac{(f_{c0} - f_{cn})}{f_{c0}} \times 100\% \tag{S3}$$

Δf_c—Rate of loss of compressive strength of concrete at the end of n test cycles (%), accurate to 0.1;

f_{c0}—Initial compressive strength of concrete specimen(MPa);

f_{cn}—Compressive strength of concrete specimen after n test cycles (MPa).

(3)Dynamic elastic modulus loss rate

For each group of three concrete specimens, use the dynamic elasticity meter for measurement. Each measurement should be repeated more than two readings, when the difference between two consecutive measurements does not exceed 0.5% of the arithmetic average of the two measurements, take the arithmetic average of the two measurements as the specimen's fundamental frequency vibration frequency.The dynamic elastic modulus was obtained according to Equation S4.

$$E_d = 13.244 \times 10^{-4} \times WL^3 f^2 / a^4 \tag{S4}$$

E_d—Modulus of dynamic elasticity of concrete(GPa);

a—Side length of square cross-section specimen(mm);

L—Length of specimen(mm);

W—Mass of specimen (kg), accurate to 0.01 kg;

f—Frequency of fundamental frequency vibration during transverse vibration of the specimen (Hz).

The change in the dynamic elastic modulus of a concrete specimen during the test is represented by the rate of loss of dynamic elastic modulus. This can be calculated using Equation S5.

$$\Delta E_r = \frac{(E_0 - E_n)}{E_0} \times 100\% \tag{S5}$$

ΔE_r—Rate of dynamic elastic modulus loss (%) of concrete specimens after *n* test cycles, accurate to 0.01;

E_0 —Initial dynamic elastic modulus of concrete specimens(g);

E_n —Dynamic elastic modulus of the concrete specimen after *n* test cycles(g).
